# First evaluation of an app to optimize and organize the processes and assessments in dental clinical courses

**DOI:** 10.1186/s12909-022-03945-z

**Published:** 2022-12-16

**Authors:** Janosch Goob, Philipp Possert, Markus Klören, Kurt Erdelt, Jan-Frederik Güth, Daniel Edelhoff, Anja Liebermann

**Affiliations:** 1 Department of Prosthetic Dentistry, University Hospital, LMU Munich, Goethestrasse 70, 80336 Munich, Germany; 2 Dentist, MCLINIC, Am Schützeneck 8, 81241 Munich, Germany; 3 Director and Chair, Department of Prosthetic Dentistry, Center for Dentistry and Oral Health, Goethe-Universität Frankfurt am Main, Theodor-Stern-Kai 7, 60596 Frankfurt am Main, Germany; 4 Polyclinic of Prosthetic Dentistry, University of Cologne, Faculty of Medicine and University Hospital Cologne, Kerpener Strasse 32, 50931 Cologne, Germany

**Keywords:** App, Mobile application, Assessment, Self-assessment, Educational technology, Organization, Dental clinical course

## Abstract

**Background:**

Digital teaching and learning tools, such as computer/mobile apps, are becoming an important factor in modern university education. The objective of our study was to introduce, analyze, and assess an organization and dual assessment app for clinical courses in dental medicine.

**Methods:**

This was a survey-based study of dental students from the clinical study phase (4th/5th year; 8th/10th semester) of a department of prosthetic dentistry at a German university hospital about the benefits of a novel web-based and mobile app for organization and dual assessment of dental clinical courses. A total of eight questions were answered in an anonymous online survey. Data were analyzed using the Kolmogorov-Smirnov test, followed by an exploratory data analysis (α < 0.05).

**Results:**

The app was given an average grade of 2.4, whereby 56.9% of the respondents rated the app with a grade of 2 (2 = good). In all, 94.6% of the study participants had not experienced any technical problems when using the app. Concerning the assessment, teaching doctor assessment (51.5 [IQR: 44.0]) was rated significantly better (*p* = 0.002) than self-assessment (39.5 [IQR: 32.8]).

**Conclusions:**

This investigation evaluated a newly introduced app to optimize dental clinical course workflows and assessment. The organizational feature was rated as good, while the daily self- and teaching doctor assessments were evaluated as less important. The results outline how the use of app technologies can provide an infrastructure for managing organization and daily assessments in dental education.

## Background

The rapid technological development in the field of digital teaching, which was further accelerated by the SARS-CoV-2 pandemic [1, 2], has made an important contribution to the evolution of medical education [3]. The development of new organizational tools, such as applications (apps), has a notable benefit for university teaching. Mobile devices, such as smartphones and tablets, have become indispensable to the 14- to 49-year-old age group, with a user share of over 95% in Germany [4]. Nevertheless, according to the UN, 37% of the world’s population had never used the internet as of 2021 [5]. During the COVID-19 pandemic, the shift from analog to digital was practically universal, and the internet has become a vital necessity for working, learning, accessing basic services, and keeping in touch [6]. Textbooks exist as e-books, lectures are available in synchronous (e.g., ZOOM, Livestream) or asynchronous (e.g., podcasts, videos) formats [2], and pharmacopoeias are web-based and mobile apps from app stores [3, 7]. With this change, conventional education has become more diverse through the implementation of digital approaches.

Traditional education in dental medicine, the organization of preclinical and clinical courses, and patient scheduling take place mainly in universities. Scheduling is more complex in dental education than in many other fields or subjects due to the clinical patient treatment semesters (4th to 5th year; 7th to 10th semester in Germany) and daily clinical dental courses in the former, whereas the latter consist mostly of fixed schedules of lectures and internships. In dental clinical courses, appointments with patients are made by the dentistry students themselves, which means that the supervising teaching doctors, who are practicing the profession in addition to their teaching roles, do not receive automatic appointment confirmations from the students. For a long time, this information was communicated by the students via direct consultation or e-mails with the supervising teaching doctor, or the students added their scheduled appointments to a list posted on the organization bulletin board. In dental clinical courses, each day has a different workload, which can change spontaneously due to appointment cancellations or last-minute appointment confirmations by patients. These spontaneous changes cannot be communicated in the conventional way and do not reach the responsible teaching doctor. To provide easier and faster organization and communication, digital items such as apps could help to replace the conventional system and adapt it to the digital age.

As empirical educational research indicates, self- and teaching doctor assessments are one of the most effective tools to support the learning and development processes [8]. Feedback and assessment in particular are key factors for both lecturers and students to further develop and improve their own skills and teaching qualifications [9, 10]. In everyday university teaching, students often only receive summative assessments by way of examinations at the end of the semester [11]. Continuous feedback and formative assessment make it possible to recognize one’s own learning progress in a more systematic way, to reflect on the learning process, and possibly to play an active role in shaping it [11, 12]. Most students appreciate continuous feedback, and it is usually the major focus for students and the motivating force for them to engage in the learning process to steadily improve and reflect their performance [11].

To simplify and digitize the above-introduced criteria, organization, and assessment in clinical dental courses, the present investigation presented a simple but progressive app to assist students. This app was implemented to make the switch from conventional to digital, to provide an infrastructure for organization and time-based scheduling for patient treatment in dental clinical courses, and to make it digitally accessible. In addition, the app provides a daily dual assessment function to assess daily treatment steps for a direct response. After completing daily treatment, students assess themselves on various criteria. Once the students have assessed themselves, the teaching doctor assesses the student on the same criteria without having seen the students’ self-assessments beforehand. These data can provide important information about students’ current knowledge, practical skills, and self-critical reflection.

Therefore, this study aimed to evaluate the specific advantages and functionality of the app and the dual assessment tool, as well as the level of student and teaching doctor satisfaction with them. To the best of the authors’ knowledge, no app-based organization and dual assessment tool has been introduced and assessed to date.

The first hypothesis of this investigation is that digital technologies such as apps are positively received by students and can support organizational processes in dental clinical courses. The second and third hypotheses are that self-assessment by the students and teaching doctor assessment by the teaching doctor, respectively, are helpful for students to receive immediate feedback regarding their daily clinical performance.

## Methods

The study was approved by the ethics committee of the Medical School (Project No. 21–0395) and declared harmless.

The web-based and mobile app “digital course organizer “(DCO) for mobile devices was developed in the Department of Prosthetic Dentistry in cooperation with an external software engineer. The app can be obtained for free via a download link and was only accessible to students and teaching doctors of this specific study. The app is available both as a web-based app and for mobile download. There is a separate interface for students and teaching doctors after the personal login. Students from the 4th and 5th years (8th and 10th semesters) can access and manage this digital platform via the web address, whereas teaching doctors use an iPad (Apple iOS, Apple iPad mini 4) to interface and manage the app. Teaching doctors are graduated dentists who are practicing the profession and also playing a teaching role to students of dentistry. Instructional videos in screencast format for interface use of the app were made available to students via an already existing course management system and online learning platform (Moodle, Moodle Pty Ltd., West Perth, Australia). An instructional presentation on how to access and use the iPad and the app was given to the teaching doctors by the software engineer. To ensure that the evaluation was as consistent as possible, the teaching doctors were guided and calibrated by the senior physicians at the beginning of each semester. In case of technical difficulties, problems, or general questions regarding the app, the students could contact the help information email address or the responsible teaching doctor.

The app is intended to help students simplify and digitize their scheduling of clinical courses. The DCO is meant to assist students in organization, scheduling of treatment dates, organization and monitoring of the progress of treatment steps, and updating of daily performance documentation, in addition to student self-assessment and assessment of students by teaching doctors during daily dental clinical courses. The app is equipped with a calendar with implemented holidays and a preset treatment schedule.

**Fig. 1:**
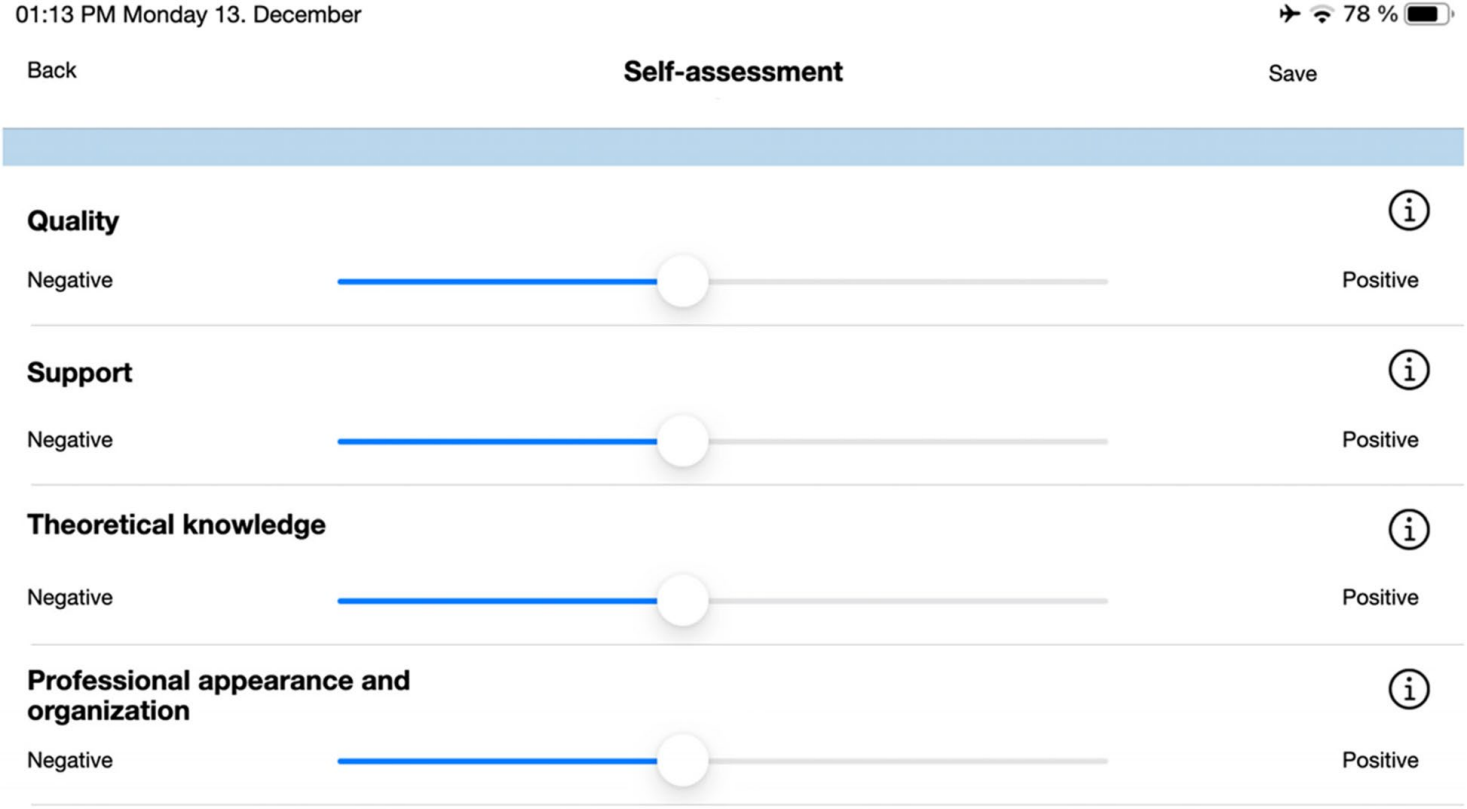
Self- and teaching doctor-assessment feature. Information button explains the question in detail

**Fig. 2:**
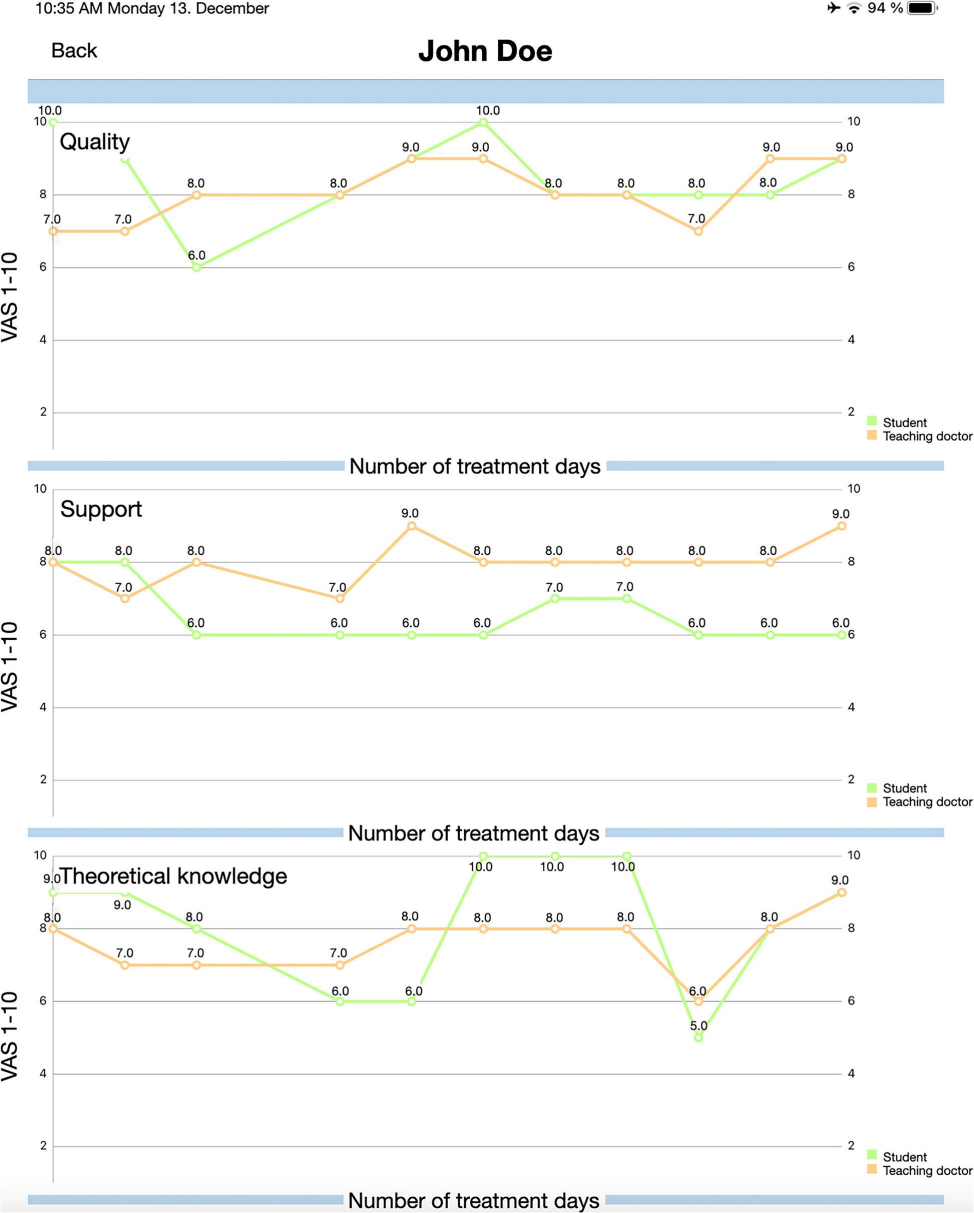
Graphical illustration of self (green; student)- and teaching doctor (orange) assessment for each day of treatment in clinical dental courses

Regarding the dual assessment, the following criteria were assessed after each treatment session: i. quality of treatment; ii. support from the teaching doctor; iii. Theoretical knowledge preparation; and iv. professional appearance and organization (Fig. 1). Students should complete the self-assessment within 24 hours. After the student self-assessment, the teaching doctors assessed the students using the same evaluation points without knowing how the students had evaluated themselves to avoid bias. Likewise, the students and teaching doctors should complete the assessment within 24 hours. After the assessment was completed, the student and the teaching doctor had access to a graphical analysis of the assessment (Fig. 2). The dual assessments were not performed blindly, but the assessment was only visible to the individual student and the supervising teaching doctor. In the case of a large discrepancy between the self- and teaching doctor assessments, the assessment was discussed with the student in order to use the daily assessment to help them to better identify and reflect on their weaknesses and strengths. The collected data are stored in an in-house server and can be managed via a separate administration app.

### Features of the “DCO” for students

**Fig. 3:**
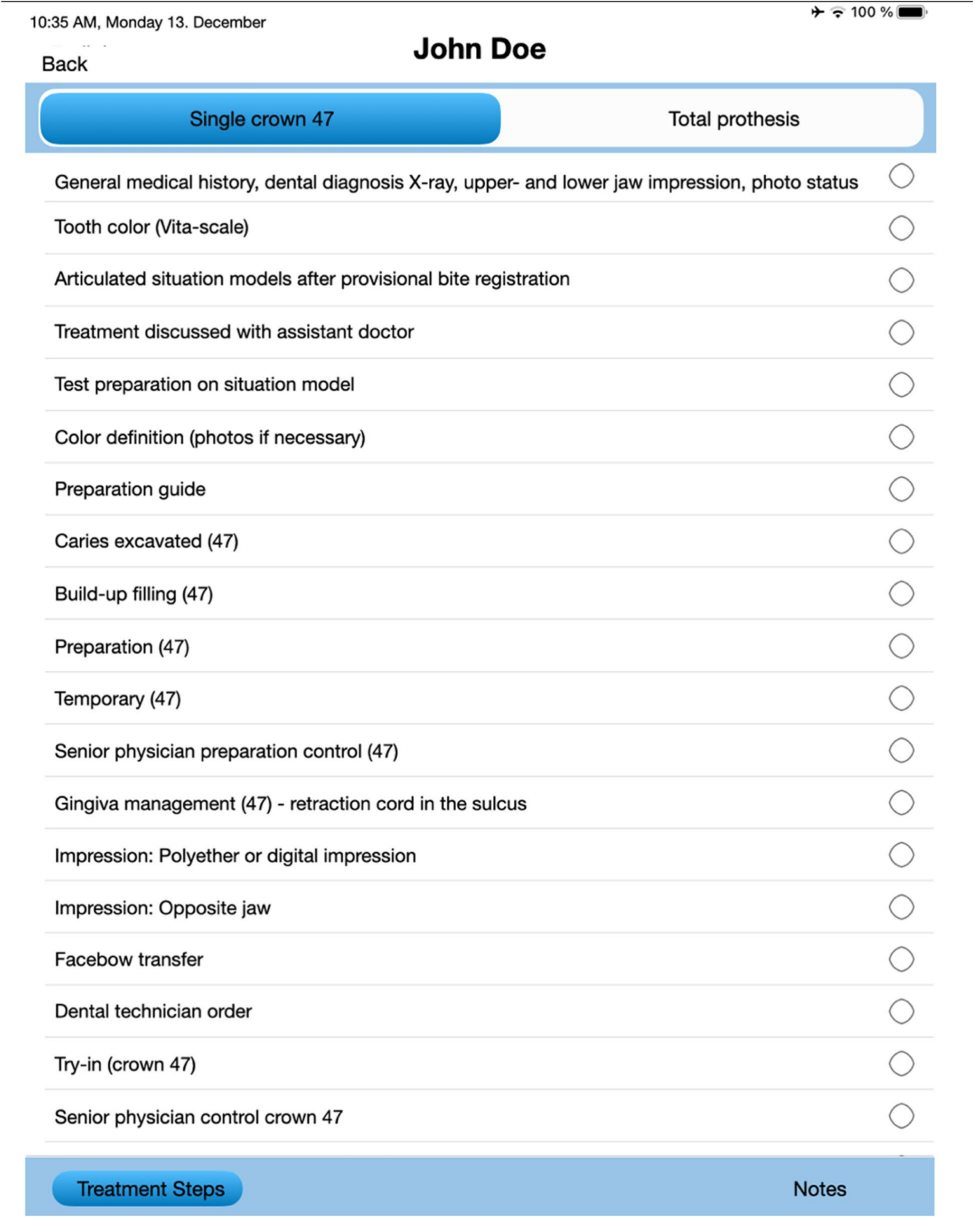
Treatment sheet with individual treatment steps. Completed treatment steps can be highlighted with a check mark by student and clarified for teaching doctor

Dental students treat their own prosthodontic patient cases under the supervision of a teaching doctor and senior physician. Each student has access to the web-based app and can use the following features:

1. Creation of a new patient file with associated planned prosthodontic care
2. Treatment sheet with individual treatment steps (Fig. 3)
3. Appointment booking and timing of treatment
4. Note function for storage of additional important information
5. Cancellation of appointments in case of cancellation by patient, illness, or incorrect booking or without reason
6. Self-assessment after daily dental clinical courses (Fig. 1)
7. Viewing divergence and direct feedback in a graphical analysis after self- and teaching doctor assessment (Fig. 2)

### Features of the “DCO” for teaching doctors

**Fig. 4:**
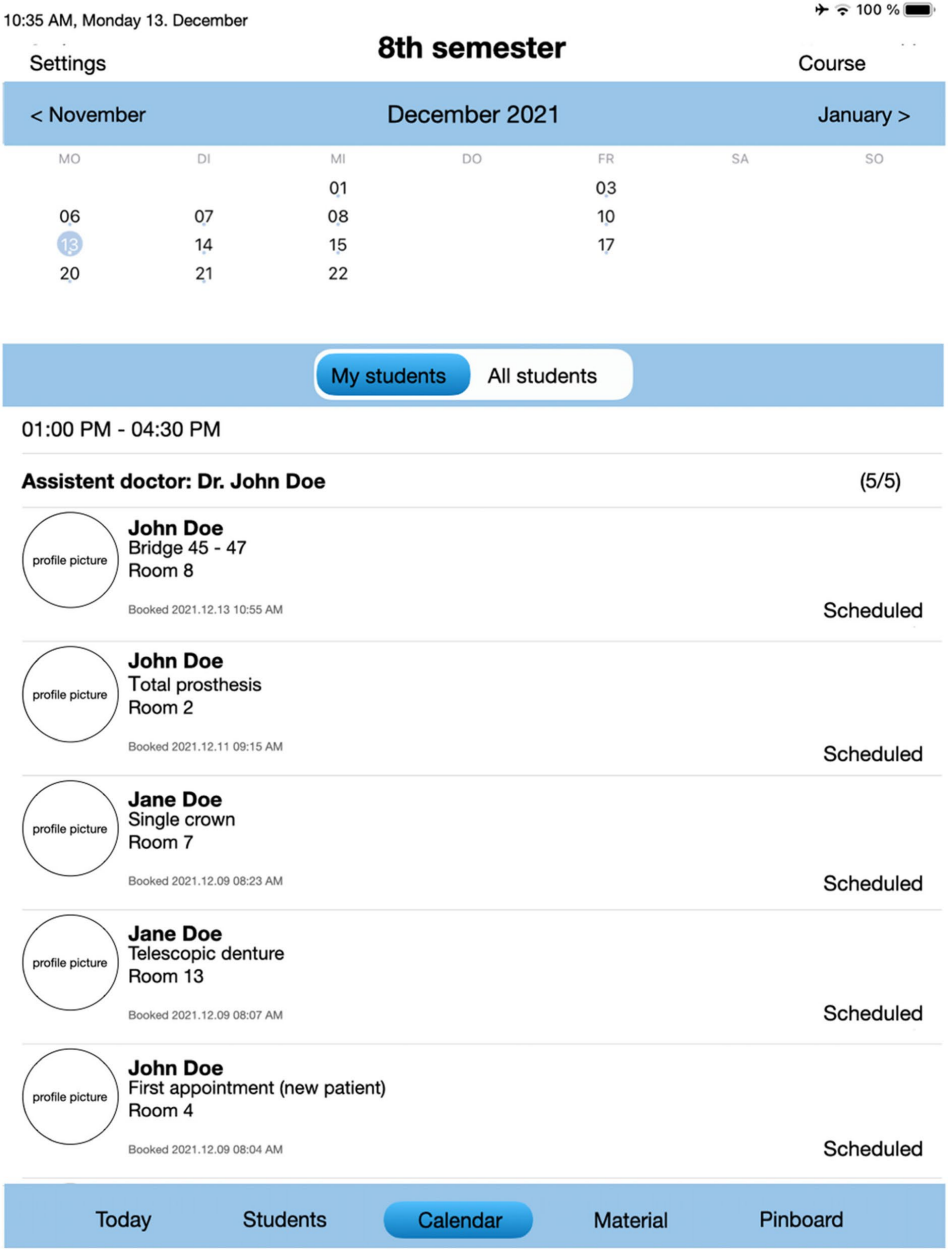
Overview appointments booked by the students from teaching doctor’s iPad interface

Each teaching doctor had their own iPad mini 4 with the application and the following features:

1. Overview of the treatments booked on each day (Fig. 4)
2. How many students are treating at the same time and in which treatment room
3. The current status of treatment
4. Overview of what the student has planned for the following treatment session
5. Cancellation of appointments in case of cancellation by the patient, illness, or incorrect booking or without reason
6. Note function to store additional important information
7. Direct assessment tool for teaching doctor assessment and graphical analysis (Fig. 2)

### Evaluation

Approximately 3 months after the course had implemented the app, an evaluation form was mailed to the students. A total of 101 students had used the app and were invited for an online evaluation. The questionnaire was created using an online survey platform called Questionstar (Questionstar, Hannover, Germany). The questionnaire consisted of a total of eight questions (Q), of which six were answered using a visual analog scale (VAS) and two with fixed-answer options (Table 1). The VAS ranged from 1 to 10 (0 to 100%). The link for the survey was sent to the students via their private university email account to allow them to complete the survey anonymously. Two reminder emails were sent at three-day intervals. The survey was stopped 2 weeks after it began. The students marked their answers to the VAS questions with a scroll bar on the line, which reflected the range from 0 to 100%. Both questions with fixed-answer options were marked with a click.

**Table 1:** Questionnaire to assess the digital course organizer (DCO) with all questions (visual analog scale [VAS] and fixed answers) and results used with N (number of participants), median, IQR (interquartile range), percentage of answers, maximum (max), and minimum (min) values

No.	Question	Answer possibility	N	Median	IQR	Number (%)	Min	Max
1	How satisfied were you with the “DCO”?	VAS (visual analog scale) range: 0% as not satisfied – 100% very satisfied	53	64.0	39.0		0.0	100.0
2	Did you feel the videos (screencast format) explaining how to use the “DCO” were sufficient?	VAS range: 0% as insufficient – 100% as very sufficient	47	76.0	16.5		30.0	100.0
3	How helpful did you find the daily teaching doctor assessment by the teaching doctor after dental clinical courses?	VAS range: 0% as not helpful – 100% as very helpful	57	51.5	44.0		0.0	93.0
4	How helpful did you find the daily self-assessment after dental clinical courses?	VAS range: 0% as not helpful – 100% as very helpful	58	39.5	32.8		0.0	88.0
5	How helpful did you find the “DCO” in organizing your daily dental clinical course and treatment?	VAS range: 0% as not helpful – 100% as very helpful	55	52.5	55.0		0.0	100.0
6	How helpful did you find the given treatment steps in preparing for your patient treatment?	VAS range: 0% as not helpful – 100% as very helpful	54	74.0	30.9		2.0	100.0
7	What grade do you give the “DCO”?	1. Very good (1) 2. Good (2) 3. Satisfactory (3) 4. Sufficient (4) 5. Defective (5)6. Insufficient (6)	58			1. 1.7 2. 56.9 3. 29.3 4. 6.9 5. 5.2 6. 0.0		
8	Did you have any technical problems with the “DCO” during the semester?	1. No 2. Yes	58			1. 94.6 2. 5.4		

### Statistical analysis

The questionnaires were analyzed with the statistical program SPSS 26 (IBM, Armonk, NY, USA). The normality of the distribution of answers was tested with the Kolmogorov-Smirnov test, followed by an exploratory data analysis. The Wilcoxon test was used for dependent samples. A *p*-value ≤0.05 was considered to indicate significance.

## Results

Out of the 101 students who used the app, 58 (58.6%) participated in the online survey.

Fifty percent of the analyzed data showed a deviation from the normal distribution and consequently were not analyzed parametrically. All questions with the corresponding results of the online questionnaire can be found in Table 1.

The students’ satisfaction with the use of the app DCO obtained a median value of 64.0 (IQR = 39.0). By contrast, the explanatory videos on how to use the app and how to use it at the beginning of the semester obtained the highest scores, with a median value of 76.9 (IQR = 16.5).

Teaching doctor assessment obtained a median value of 51.5 (IQR = 44.0), and self-assessment was rated lower, with a median value 39.5 (IQR = 32.8). Concerning the assessment, teaching doctor assessment (median value = 51.5 [IQR = 44.0]) was rated significantly better (*p* = 0.002) than self-assessment (median value = 39.5 [IQR = 32.8]).

Assistance with patient scheduling obtained a median value of 52.5 (IQR = 55.0), and assistance throughout the given treatment steps and the possible preparations for patient treatment obtained a median value of 74.0 (IQR = 30.9).

In general, the app was given an average school grade of 2.4, whereby 1.7% assigned a grade of 1 (very good), 56.9% a grade of 2 (good), 29.3% a grade of 3 (satisfactory), 6.9% a grade of 4 (sufficient), and 5.2% a grade of 5 (defective).

Among the students, 94.6% said they had not experienced any technical problems in using the app, in contrast to 5.4%, who had experienced minor technical problems. These problems were due to incorrect login data and accounts created incorrectly by the software engineer and were easily solved. Once students had a working account, there were no further issues.

## Discussion

Apps were originally designed for general productivity and information access. Then, rapidly increasing public demand led to an explosive growth to include other valuable categories, such as the use of mobile digital media and applications in education [3]. Besides the presentation and illustration of the new mobile-based application “DCO”, our study is, to authors´ best knowledge, the first to assess the use of this organizational tool and dual assessment application in dental education.

The survey showed mid-level acceptance among the interviewed students despite two reminders. The response rate was 58.8%, which can be classified as average but still provides a reliable answer and conclusion to the research issue [13].

The answers to Q1, Q5, and Q7 support the first hypothesis that digital applications such as apps can be integrated into dental education and facilitate organization. Student agreement and overall satisfaction with the availability of the digital organization and dual assessment application obtained a median value of 64.0% (Q1). In general, this indicates that the students are supportive of the application’s implementation in the daily treatment routine. This mirrors the fact that the number of apps offered for training and teaching in medicine is steadily increasing [14]. Students can now book appointments from home, are much more flexible in their scheduling, and have an overview of their appointments, treatment progress, and important notes stored in the app.

The explanatory videos in the screencast format were found to be good and sufficient for operating the app features (Q2). Requests to the help information email address in case of technical difficulties, problems, or general questions were almost non-existent, which is also reflected in Q8. There were only occasional incorrect appointment bookings or incorrectly created treatment step sheets, which could be corrected by the supervising teaching doctor or student.

The results of evaluations of the dual assessment feature, self-assessment, and teaching doctor assessment were significantly different (*p* = 0.002). The teaching doctors perceived a greater advantage of the teaching doctor assessment than did the students of the self-assessment, confirming the third hypothesis and rejecting the second hypothesis in our investigation. This is possibly due to the fact that it is difficult for the students to assess themselves correctly for dental steps they have never or rarely performed on patients [15]. High-performance students tend to evaluate themselves critically and more accurately and tend to underrate their performance, whereas low performers tend to overrate themselves [15–18]. In this context, the ability to self-assess one’s qualifications as an oral health care provider is an important competency [19–21]. Therefore, it will be of interest to examine the differences between self- and teaching doctor assessments in further studies.

The advantage of the app for planning and organizing the patient’s appointment calendar was rated as neutral. It is possible that students are no longer familiar with the old booking system, and many students may use their own private calendars. These observed results would likely have been more favorable if the students knew the conventional system of appointment organization.

The prefabricated flowcharts (Fig. 3) for the individual prosthetic treatments with treatment steps available in the app were found to be very helpful for preparation. A clear, predetermined structure and template, with individual customization, gives students confidence and can help them in the theoretical preparation of their practical work.

Finally, the app “DCO” was given an average score of 2.4 (1 = very good to 6 = insufficient), whereby 56.9% of the respondents rated the app with a 2 (2 = good).

This investigation presents an app for mobile devices and desktop computers to optimize clinical teaching workflows and assessment. The results outline how the use of app technologies can provide an infrastructure for managing organization and daily evaluation in dental education.

Furthermore, by switching from paper format to digital media, a sustainable benefit can be achieved in the long term. By adapting the app to other curricula, this app could also be used to organize education at other universities. Some limitations of the present investigation should be mentioned. IT support is required to update the app, back up data, and troubleshoot problems, which, in addition to the high costs of programming, leads to continuous follow-up costs. The technology and the handling of digital devices was not questioned and was assumed to be known without verification. Moreover, students without access to a private digital device might be disadvantaged. This factor could compromise the survey answer and satisfaction rates and could lead to many other inequities in the perceived experience with the app. As already mentioned, the students in this investigation were not familiar with the conventional system of appointment booking and organization, which might have biased the assessments. Generally, the evaluation is missing a control group that has never worked with the digital organization and assessment tool. By contrast, they seem to consider it as an established digital tool. A weakness of the assessment feature is that no time limit was set to finish the self- and teaching doctor assessments, which might have negatively affected the results.

After the app has been presented and assessed and the advantages and disadvantages described, it would be of great interest for educational research in general to take a closer look at the dual assessment and its results. Therefore, it would be inspiring to investigate the accuracy and discrepancy of self- and teaching doctor assessments according to academic performance in daily dental clinical courses over time. The app is currently only available for our faculty but could be made available and adapted for other universities and subject fields to enable multicenter studies. One idea for further research could be to run one course without the digital organization with the same students/participants or to divide a semester into users and non-users and compare the results. It would also be interesting to analyze the use of the app for a longer period.

## Conclusion

The implementation of a novel mobile app centered on the organization and assessment of the daily dental clinical courses in dental education was well received by students of the department of prosthetic dentistry. The results outline how the use of app technologies can provide an infrastructure for managing the organization of the clinical dental courses and daily assessment in dental education. The organizational features were evaluated as beneficial, while the daily self- and teaching doctor assessments were rated as less important.

Within the limitations of this investigation, these data suggest that app-based organization of dental clinical courses may have the potential to streamline enhanced management in education. Whether the dual assessment is beneficial to education must be clarified in a further investigation. Apps that can improve networking, organization, and efficiency in university education will be a vital educational advantage in the future.

## Data Availability

The datasets generated and analyzed during the current study are not publicly available due to the fact that all essential data are already included in the manuscript but are available from the corresponding author on reasonable request.

## Abbreviations

DCO: Digital course organizer
Q: Question
VAS: Visual analog scale
IQR: Interquartile range
